# Connect to Protect: Dynamics and Genetic Connections of Highly Pathogenic Avian Influenza Outbreaks in Poultry from 2016 to 2021 in Germany

**DOI:** 10.3390/v14091849

**Published:** 2022-08-23

**Authors:** Jacqueline King, Christoph Staubach, Christiane Lüder, Susanne Koethe, Anne Günther, Lina Stacker, Dennis Rubbenstroth, Klaas Dietze, Christian Grund, Franz J. Conraths, Timm Harder, Martin Beer, Anne Pohlmann

**Affiliations:** 1 Institute of Diagnostic Virology, Friedrich-Loeffler-Institut, 17493 Greifswald-Insel Riems, Germany; 2 Institute of Epidemiology, Friedrich-Loeffler-Institut, 17493 Greifswald-Insel Riems, Germany; 3 Institute for International Animal Health/One Health, Friedrich-Loeffler-Institut, 17493 Greifswald-Insel Riems, Germany

**Keywords:** highly pathogenic avian influenza viruses, H5N8, H5N5, clade 2.3.4.4, phylogenetic network analyses, next-generation sequencing, MinION, epidemiology

## Abstract

During autumn/winter in 2016–2017 and 2020–2021, highly pathogenic avian influenza viruses (HPAIV) caused severe outbreaks in Germany and Europe. Multiple clade 2.3.4.4b H5 HPAI subtypes were responsible for increased mortality in wild birds and high mortality and massive losses in the poultry sector. To clarify putative entry sources and delineate interconnections between outbreaks in poultry holdings and wild birds, we applied whole-genome sequencing and phylodynamic analyses combined with the results of epidemiological outbreak investigations. Varying outbreak dynamics of the distinct reassortants allowed for the identification of individual, putatively wild bird-mediated entries into backyard holdings, several clusters comprising poultry holdings, local virus circulation for several weeks, direct farm-to-farm transmission and potential reassortment within a turkey holding with subsequent spill-over of the novel reassorted virus into the wild bird population. Whole-genome sequencing allowed for a unique high-resolution molecular epidemiology analysis of HPAIV H5Nx outbreaks and is recommended to be used as a standard tool. The presented detailed account of the genetic, temporal, and geographical characteristics of the recent German HPAI H5Nx situation emphasizes the role of poultry holdings as an important source of novel genetic variants and reassortants.

## 1. Introduction

Recurrent epidemic outbreaks of highly pathogenic avian influenza (HPAI) in Europe affected both poultry and wild bird species alike and entailed high mortality and devastating (economic) losses [1]. Rooted in the A/Goose/Guangdong/1/1996 (gs/GD) lineage first detected in China in 1996 [2] and catalyzed by the co-circulation of low pathogenic avian influenza viruses (LPAIV), multiple HPAI H5Nx subtypes and reassortants were identified in Europe in 2016/17 and 2017/2018 [3,4,5,6,7,8]. In winter 2020/2021, novel HPAIV H5N8 reassortants emerged in Europe that again caused high mortality in wild birds and affected poultry holdings [9,10].

HPAI H5 viruses of the respective gs/GD lineage have been circulating endemically among poultry in East and Southeast Asia since 2003 [11,12,13]. Spill-over events into the wild bird population initiated the subsequent transcontinental spread by migratory birds [14,15]. From late 2013 onwards, HPAIV carrying H5 hemagglutinins of clade 2.3.4.4 combined with varying neuraminidase segments (H5N8, H5N5, H5N6) were identified and continue to circulate in Southeast Asia [16,17]. Clade 2.3.4.4 H5 viruses, known for their unprecedented tendency for frequent reassortment, have further evolved until 2016/17 to cluster within four genetic groups, termed A to D [18]. H5N8 viruses belonging to group A (clade 2.3.4.4a) were responsible for the 2014/2015 HPAI outbreaks in Central Asia, Russia, North America, and Europe [12,18,19,20]. After the transcontinental group A outbreaks, the genetically differing group B of clade 2.3.4.4 (clade 2.3.4.4b), commonly known as “Gochang-like”, was identified in wild waterfowl in Southeast Asia in May 2016 at known breeding and molting areas along the Russian–Mongolian and Russian–Chinese border, for example at Lake Uvs Nuur, Russia, and Qinghai Lake, China [21,22,23]. Subsequently, clade 2.3.4.4b HPAIV H5Nx spread across most European countries from fall 2016 onwards, causing frequent outbreaks in poultry holdings and increased mortality in the wild bird population [3,7,24,25,26]. In 2020, numerous outbreaks in wild birds were preceded by the detection of H5N8 viruses in poultry in Iraq in May 2020, in Russia in July and August 2020, and in Kazakhstan in September 2020. The analysis of the genomes revealed the connection of these viruses to outbreaks in migratory waterfowl in Germany in 2020 [9,10].

The role of wild migratory birds in the dissemination of AIV has been thoroughly investigated for previous HPAIV H5Nx outbreaks in Europe [11,14,15,18,21,22,27,28,29,30,31,32,33,34]. Within the 2016/2017 HPAI epidemic, multiple independent swarm incursions of clade 2.3.4.4b HPAIV H5Nx by migratory birds led to the detection of two subtypes (H5N8, H5N5) encompassing five distinct reassortants in Germany [1,7,8], individually displaying differing outbreak dynamics. Within the 2020/2021 epidemic, even more distinct reassortants of clade 2.3.4.4b HPAIV viruses were detected, summing up to five different subtypes (H5N8, H5N5, H5N1, H5N4, and H5N3) with seven genotypes [10].

Although studies have previously been conducted for multiple European countries [3,11,24,26,33,35,36,37], detailed insights into the connections between wild birds and poultry holdings, in addition to the role of poultry production in the dissemination and progression of HPAIV H5Nx in Germany, have yet to be analyzed and evaluated.

Here we present a detailed analysis of German poultry outbreaks using phylogenetic networks, time-scaled phylogeographic analyses, and epidemiological outbreak investigations. AIV genomes sampled from poultry outbreaks and a respective subset of wild bird cases generated a foundation for a full connection network between the individual outbreaks revealing insights into the role of poultry as an important source of novel genetic variants and reassortants.

## 2. Materials and Methods

### 2.1. Definitions—Outbreaks, Cases, Clusters, and Hotspots

For the sake of common understanding, the definition of certain terms must be solidified from here on. Outbreaks are seen as the infection of one holding, affecting at least one animal. Thus, every afflicted poultry holding stands as an individual outbreak. As wild birds are usually recorded singularly, each infected wild bird is defined as a case.

The term cluster is defined here as groups of more than four poultry outbreaks with short phylogenetic distances of >99.8% sequence identity. A poultry hotspot describes a geographic area of high commercial poultry holding density.

### 2.2. Sample Selection and RNA Extraction

Viral RNA extracted from tracheal or cloacal swabs and organ samples tested positive for HPAIV H5Nx by RT-qPCR methods in the National Reference Laboratory for AIV (Friedrich-Loeffler-Institut, Greifswald—Insel Riems, Germany [38]).

A representative subset of RT-qPCR-positive samples was selected for further sequencing, with at least one sample per poultry holding analyzed in all possible cases. In outbreaks of particular interest, multiple samples per holding were sequenced. Wild bird cases were selected to cover all federal states in Germany, a wide range of species, and the full study period. In addition, wild bird cases in close proximity to poultry outbreak clusters were likewise selected for sequencing.

RNA was extracted by using Trizol LS (ThermoFisher Scientific, Waltham, MA, USA) in combination with the QIAamp Viral RNA Mini Kit (Qiagen, Hilden, Germany) according to the manufacturer’s instructions.

### 2.3. IAV-End-RT-PCR

Utilizing an influenza A virus (IAV) universal segment amplification protocol [39], the RNA was amplified with Invitrogen Superscript III One-Step RT-PCR and Platinum Taq (ThermoFisher Scientific) with one primer pair (Pan-IVA-1F: TCCCAGTCACGACGTCGTAGCGAAAGCAGG; Pan-IVA-1R: GGAAACAGCTATGACCATGAGTAGAAACAAGG), binding to the conserved ends of the IAV genome segments. After purification of the PCR products with AMPure XP Magnetic Beads (Beckman-Coulter, Brea, CA, USA), the RT-PCR amplicons were subsequently sequenced by Sanger or next-generation sequencing (NGS) on the Ion Torrent (ThermoFisher Scientific) or MinION (Oxford Nanopore Technologies, ONT, Oxford, UK) platform as previously described [1,7,40].

### 2.4. Next-Generation Sequencing and Consensus Generation

The NGS protocol for the IonTorrent platform (ThermoFisher Scientific) started with the fragmentation of the RT-PCR products on a Covaris M220 Ultrasonicator (Covaris Ltd., Brighton, UK), obtaining a target size of 500 bp. In the following library preparation step, the sonicated cDNA was further processed with the GeneRead DNA Library L Core Kit (Qiagen) and IonTorrent Ion Xpress Barcode Adapters (ThermoFisher Scientific) to create end-repaired and barcoded cDNA fragments. Size selection of the libraries was completed by utilizing AMPure XP Magnetic Beads (Beckman Coulter, Fullerton, CA, USA). The finished libraries were subsequently quality checked with High Sensitivity DNA chips and reagents on a Bioanalyzer 2100 (Agilent Technologies, Böblingen, Germany) and quantized via quantitative PCR with the KAPA Library Quantification Kit IonTorrent (Roche, Mannheim, Germany). Sequencing was conducted on an IonTorrent S5XL (ThermoFisher Scientific) in combination with the OneTouch 2 System (ThermoFisher Scientific) to produce approximately 500,000 reads per sample.

After the quality check, trimming, and screening for adapter and primer contamination, the produced raw data were used to assemble full-genome consensus sequences ready for further genetic analyses. This was achieved in a de novo assembly (Geneious Mapper) and iterative map-to-reference approach employing Bowtie2 (v.2.3.0; [41]) in the Geneious Prime Software Suite (v2020.0, Biomatters, Auckland, New Zealand).

For the MinION platform (Oxford Nanopore Technologies, ONT, Oxford, UK), a one-step library protocol was used (Rapid Barcoding Kit (SQK-RBK004, ONT) for transposon-based library preparation and multiplexing. Sequencing was directed according to the manufacturer’s instructions with an R9.4.1 flow cell on Mk1C device with MinKNOW Software (ONT, UK), including live basecalling of the raw data with Guppy (ONT, UK) and demultiplexing, quality check and trimming step to remove low quality, primer and short (<50 bp) sequences. After sequencing, full-genome consensus sequences were generated in a map-to-reference approach utilizing MiniMap2 [42]. As reference genomes, a curated collection of all HA and NA subtypes alongside an assortment of internal gene sequences was chosen to cover all potentially circulating viral strains. Polishing of the final genome sequences was performed manually after consensus production according to the highest quality (60%) in Geneious Prime (Biomatters, Auckland, New Zealand).

### 2.5. Phylogenetic and Phylogeographic Analysis

Segment-specific trees were generated with RAxML [43] utilizing the nucleotide model GTR GAMMA with rapid bootstrapping and search for the best-scoring maximum likelihood tree together with 1000 bootstrap replicates or with FastTree [44]. Subsequently, the obtained segment-specific trees underwent network analysis in Splitstree4 [45] for the generation of a final consensus tree comprising all IAV gene segments. Further phylogenetic network analyses were conducted for each genotype after concatenation and alignment of full-length nucleotide sequences of the eight gene segments in Geneious Prime (v.2020.0, Biomatters). Median-joining networks were subsequently generated and displayed in the software program SplitsTree4 [45] with 2000 spring embedder iterations, excluding gapped sites and scaling nodes by taxa. Time-scaled trees of the concatenated alignments of each genotype were calculated with BEAST (v.1.10.4) software package [46] using a GTR substitution model with Gamma distribution, an uncorrelated relaxed clock with a lognormal distribution, and coalescent constant population tree models. Phylogeographic continuous trait spatial diffusion models were calculated for the genotype tree sets using a Bayesian SkyRide coalescent model with latitude and longitude of the sampling locations and random jitter of 0.0001 for identical coordinates. Chain lengths were set to 50 million iterations, and convergence was checked via Tracer (v.1.7.1). Time-scaled summary maximum clade credibility trees (MCC) with 10% post-burn-in of each genotype were created using TreeAnnotator (v.1.10.4) and visualized with FigTree (v.1.4.4). The MCC trees show estimates of the time and their 95% highest posterior density (HPD) confidence intervals at each node. The spatiotemporal diffusion models were analyzed and visualized using Spread (v.1.0.7) [47] and QGIS (v.3.16, QGIS.org). Geographical geojson vector maps were created (http://opendatalab.de/projects/geojson-utilities/) (accessed on 1 March 2021) with open data provided by the Federal Agency for Cartography and Geodesy (https://gdz.bkg.bund.de/) (accessed on 1 March 2021).

Epidemiological data, including outbreak date, affected species (poultry and wild birds), holding type and size, location of outbreak (poultry holding), or case (wild bird discovery site), were extracted from the German Animal Disease Notification System (TSN—*Tierseuchennachrichten*). Additional epidemiological data were extracted from epidemiological outbreak investigation reports and official reports from the German Federal Ministry of Food and Agriculture (BMEL—*Bundesministerium für Ernährung und Landwirtschaft*).

## 3. Results

### 3.1. Background Information

Starting in early November 2016, the first cases of the HPAI H5Nx in the 2016–2017 season were detected simultaneously in diving duck species (*Anseriformes* sp.) in the north (Lake Plön) and south (Lake Constance) of Germany [1]. The events persisted until August 2017 after peaking during the winter of 2016/17, with more than 1100 cases identified in wild birds, 92 outbreaks in poultry holdings, and 15 outbreaks in captive birds from zoos/wildlife parks. Small (backyard) and large commercial poultry holdings of different species, age groups, and type of stock were affected [1]. In 2017/18, 2018/19, and 2019/2020, only limited case numbers in wild birds and few outbreaks, mainly in small commercial poultry holdings, were detected in Germany [8,48,49]. Starting in late October 2020, multiple cases of H5Nx HPAI cases were detected mainly in the North-German coastal regions leading to massive die-offs in wild birds, especially affecting birds of the orders *Anseriformes* and *Charadriiformes*. The events continued until August 2021, with a peak during the winter months. Over 1300 wild bird cases and more than 250 outbreaks in poultry holdings (mainly turkey and layer chicken holdings) pointed again to the necessity to examine the role of poultry holdings and the poultry-wild bird interface in the HPAI epidemic.

In season 2016/2017, five reassortants belonging to subtypes H5N5 and H5N8 were identified, all linked by a shared HA segment rooted in the 2.3.4.4b lineage and individually distinguished by diverse reassorted segments. In 2020/21, seven reassortants belonging to subtypes H5N8, H5N5, H5N1, H5N4, and H5N3 were detected. Table 1 summarizes the metadata of the sequenced genomes. To conduct phylogeographic analyses, samples were sequenced to cover all obtainable poultry outbreaks (104 genome sequences) and backed with sequences from captive zoo bird or wild bird cases (38 genome sequences) from the 2016/17 events. During 2020/21, multiple reassortants were identified with H5N8 dominating the situation and being the main causative agent in large poultry holdings and, therefore, target for the outbreak analyses and follow-up with 122 genome sequences from poultry holdings covering a representative set and backed with sequences from captive zoo bird outbreaks or wild bird cases (39 genome sequences).

**Figure 1:**
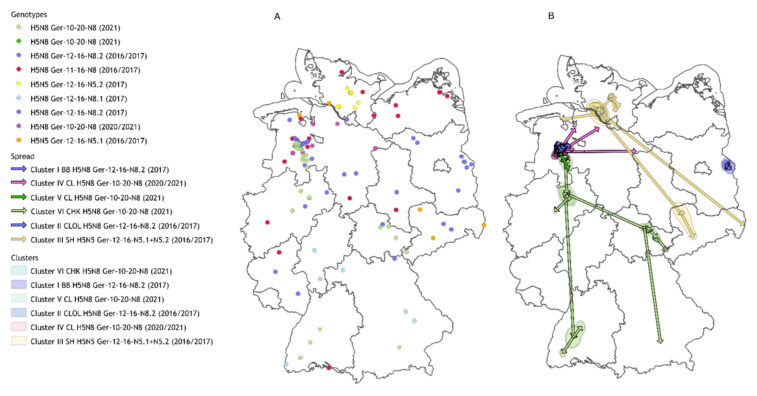
Geographic overview, genotype assignment, and connected clusters of HPAIV genomes from 2016/2017 and 2020/2021 in Germany. Panel (**A**): Sequenced outbreaks and cases with their assigned genotype (circles). Panel (**B**): Identified connected cluster areas (polygons) with their directed spread (arrows). BB—Brandenburg, CHK—Chicken, CL—Cloppenburg, OL—Oldenburg, SH—Schleswig-Holstein.

Although all reassortants were described in wild birds and poultry outbreaks during the two periods, they can be clearly discriminated by their putative genetic origin and their distinct spread and distribution patterns: (i) Ger-11-16-N8—H5N8, entry from Northeast Europe with subsequent spread along the Northern coastline of Germany, mainly small backyard holdings affected; (ii) Ger-12-16-N8—H5N8, entry from Central East Europe and dissemination throughout Central Germany, detection of large poultry clusters with an indication of direct connections; (iii) Ger-12-16-N5—H5N5, entry via Central/Southeast Europe with limited spread; (iv) Ger-10-20- N8—H5N8 Entry from Central Asia and Northern Europe and spread along the Northern coasts in Germany with subsequent dissemination in Central Germany in large connected poultry clusters (Figure 1, Table 1).

The time and location of reassortment of these viruses and subsequently possible entry points into Europe have been investigated by Lycett et al. [34]. Reassortment designation and genotype assignment was performed as previously described [7,10]. A joint analysis of the local German virus sequences with a set of international sequences (analysis available in the https://zenodo.org/ repository under DOI 10.5281/zenodo.6826696) underlines the clustering of the proposed groups, shows internationally occurring reassortants, and does not suggest the involvement of viruses from international poultry outbreaks in German outbreak clusters.

### 3.2. Direct Wild Bird Introductions and Spread—H5N8 Ger-11-16 and Ger-12-16.1

#### 3.2.1. H5N8 Ger-11-16

##### Epidemiological Data

The initial HPAI H5N8 reassortant identified from November 2016 to August 2017 in Germany, designated Ger-11-16 in accordance with Pohlmann et al. [7], was first detected in wild diving duck species on 7 November 2016 at Lake Plön (Schleswig-Holstein) and Lake Constance (Baden-Wuerttemberg) [1]. At the outset, Ger-11-16 continued to spread along the Baltic and the North Sea coastline before later moving inland. Of the total 142 sequenced samples, 25 Ger-11-16 outbreaks were identified in domestic poultry, and nine Ger-11-16 sequences were derived from wild bird cases (Table 1).

##### Phylogenetic Analysis of Ger-11-16

Phylogenetic median-joining network analysis was performed to study the genetic relations between Ger-11-16 viruses (Supplementary Figure S1). Significant for Ger-11-16, the analysis revealed several connections between wild bird cases and poultry outbreaks, as well as, in individual cases, relations among backyard poultry holdings in Northern Germany. No clusters were established with concatenated full-length nucleotide sequences of Ger-11-16 ranging from 98.97% to 99.99% identity.

##### Wild Bird-Mediated Transmissions of Ger-11-16

The first wild bird case detected in Northern Germany (AR8444/16—Supplementary Figure S1, red star) was genetically highly similar (>99.9%) to a small commercial poultry outbreak (AR8595/16—red star) sampled two days later in approximately 70 km distance, sharing a predicted common ancestor (Supplementary Figure S1). Two further backyard holdings (AR8758/16, AR8790/16—orange star), simultaneously reported, but with approximately 200 km distance between both holdings, likewise shared a predicted common ancestor and high identity levels of >99.9%. Likewise, neighboring backyard holdings (AR9311/16, AR9528/16—Supplementary Figure S1, green star) in Mecklenburg-Western Pomerania (approximately 100 m distance) reported in a two-day interval shared a predicted common ancestor in the phylogenetic analysis, suggesting separate entries by infected wild birds (Supplementary Figure S1). These findings suggest the circulation of Ger-11-16 in the Northern wild bird population and subsequent individual introductions into poultry holdings.

##### Farm-to-Farm Transmissions within Ger-11-16

Evidence of direct farm-to-farm virus transmissions was scarce, and no clusters were identified. The median-joining network analysis, however, suggested a close relationship between two backyard poultry holdings in Mecklenburg-Western Pomerania (holding A: AR9433/16, holding B: AR9738/16, AR9764/16—yellow star, Supplementary Figure S1). The two neighboring locations, only separated by 29 m, reported outbreaks within four days and showed >99.96% genome identity. Here, a transmission event by direct contact or mechanical/biological vectors, for example, human interaction, shared equipment/feed/bedding, fomites, or wind, is highly likely.

##### Circulation Period of Ger-11-16

The last Ger-11-16 cases in Germany were reported in two swans in August 2017 (AR3396/17, AR3397/17—Supplementary Figure S1, blue star), located in Saxony-Anhalt and sharing high sequence identity levels of 99.96%. The median-joining network analysis established a connection between both swans and a commercial chicken holding in Lower Saxony (AR11406/16—Supplementary Figure S1, blue star), affected in December 2016 in 250 km airline distance. These findings suggest the continuous circulation of HPAI H5N8 Ger-11-16 in the local waterfowl population rather than a novel introduction of the equivalent reassortant in summer 2017. The overall lack of Ger-11-16 outbreaks in large commercial poultry holdings, tight clustering, and long infection chains point toward multiple recurring entries from infected wild birds into the poultry population, affecting mainly backyard holdings in Northern Germany. This could potentially be associated with the lack of commercial poultry hotspots of high density in Northern Germany. The genetic analysis displays multiple established genetic connections between wild birds and poultry outbreaks, alongside few potential farm-to-farm transmissions.

#### 3.2.2. H5N8 Ger-12-16.1

##### Epidemiological Data, Genetic Distinction, and Phylogenetic Analysis

A further H5N8 reassortant first discovered in December 2016 in Germany, termed Ger-12-16 [7], entails two similar reassortants, Ger-12-16.1 and Ger-12-16.2, genetically differing by only their PB1 segment (Supplementary Figure S2).

The Ger-12-16.1 group comprises six wild bird cases and two poultry outbreaks, concentrating on Southern Germany with outbreaks in the federal states of Bavaria, Baden-Wuerttemberg, Hesse, Thuringia, and Saxony. All disease events were identified in January–February 2017 (Table 1). A diverse range of wild bird species was infected with Ger-12-16.1, including a great egret (*Ardea alba*), greylag goose (*Anser anser*), black swan (*Cygnus atratus*), white stork (*Ciconia ciconia*), and tawny owl (*Strix aluco*). The two poultry outbreaks comprise one commercial turkey holding and one small holding in Bavaria. Overall, the individual outbreaks demonstrate identity levels of 99.67–99.75%, and no clusters were found.

### 3.3. Cluster I and II: Cluster Development and Modification by Genetic Drift—H5N8 Ger-10-16.2

Although reassortant Ger-12-16.2 differed from Ger-12-16.1 only by the PB1 segment, it presented drastically altered outbreak dynamics in comparison, potentially due to the area of circulation and concomitant high commercial poultry holding density. This reassortant caused more than half of all outbreaks in poultry (60 outbreaks), affecting predominately turkey holdings. Further sequences were derived from 10 wild bird cases and 1 emu held in captivity. The first respective report was on 13 December 2016, in a turkey holding in Lower Saxony (AR10523/16), with ongoing records of the reassortant virus until May 2017. Overall, the activity is geographically concentrated in Central Germany and, unlike Ger-11-16 and Ger-12-16.1, two distinct poultry clusters could be pinpointed.

Median-joining networks and phylogeographic analysis allowed the examination of genetic connections within reassortant Ger-12-16.2 and ensuing detection of two poultry clusters: (i) Cluster I “Brandenburg (BB)” and (ii) Cluster II “Cloppenburg/Oldenburg (CLOL)” (Figure 1B).

#### 3.3.1. Cluster I—“Brandenburg (BB)”

**Figure 2:**
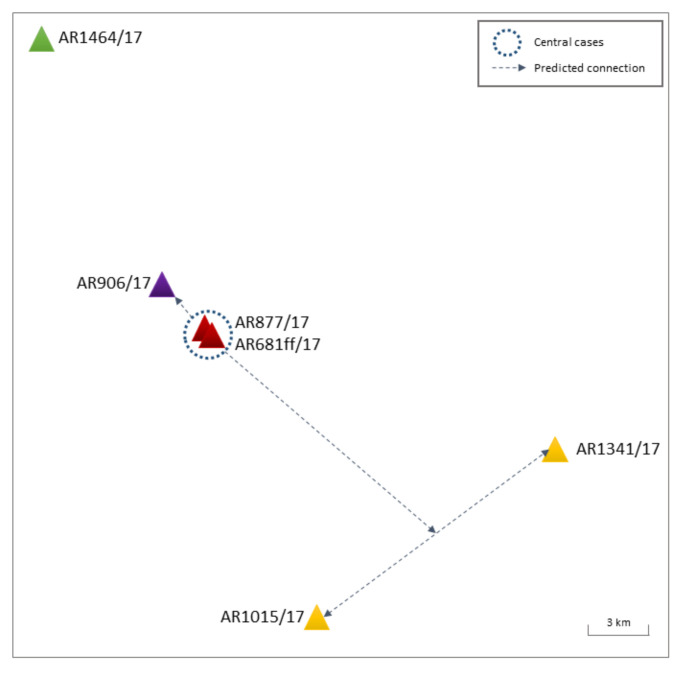
Graphic representation of Cluster I BB H5N8 in poultry holdings. Holdings are colored in red, purple, yellow, and green, as described in the main text. For reasons of anonymity, no explicit locations are shown. Dotted circles—central cases, dotted lines—predicted connections.

In total, nine poultry holdings in Brandenburg tested positive for HPAIV H5N8 during January–February 2017, all of which were examined in dedicated epidemiological field investigations. A cluster of five outbreaks (identity levels >99.8%) was reported within a 3-week period, and a 20 km radius was established (Figure 2, Supplementary Figures S2 and S3).

The first two disease events in duck fattening farms (AR877/17, AR681ff/17—Figure 2 and Supplementary Figure S2, red) in close proximity (100 m) tested positive within a 5-day period. Belonging to one operating farming group with shared vehicles and personnel, the disease events were epidemiologically classified as connected outbreaks. The generated sequences clearly confirmed this (99.94% identity, Supplementary Figure S2). A farm with breeding ducks located within a 1.2 km linear distance of the former outbreaks showed clinical symptoms one day after and subsequently tested positive for H5N8 (AR906/17—Figure 2 and Supplementary Figure S2, purple). The sequences likewise indicate a direct connection, albeit no obvious direct contacts (vehicles, personnel) could be identified in the epidemiological outbreak investigations. The short, direct distance (1.25 km), free of buildings, hills, or trees that could serve as a wind shelter, and documented wind maxima of up to 4–5 from varying directions during possible days of infection could indicate a wind-mediated transmission between these outbreaks. Two further genetically highly similar outbreaks (>99.9% identity) in a duck fattening farm (AR1015/17—Figure 2 and Supplementary Figure S2, yellow) and turkey holding (AR1341/17—Figure 2 and Supplementary Figure S2, yellow) in 32 km airline distance to one another were identified shortly after, located south and southeast of the primary outbreaks (AR877/17, AR681ff/17), respectively. Again, no direct connections between the holdings could be established by the epidemiological outbreak investigations.

In mid-February 2017, a duck breeder farm (AR1464/17—Figure 2 and Supplementary Figure S2, green) approximately 35 km north of the initial poultry outbreaks in Brandenburg (AR877/17, AR681ff/17) was confirmed positive for HPAIV H5N8. Although located at a similar distance as AR1015/17 and AR1341/17 to the primary AR877/17 and AR681ff/17 outbreaks, genetic analysis established lower identity levels of 99.6% to the previously described Ger-12-16.2 poultry outbreaks. The median-joining analysis also grouped AR1464/17 within a separate branch of the network, sharing the closest relation to a common precursor 9raylag goose (AR703/17) identified on 25 January 2017 in Lower Saxony (Supplementary Figure S2).

#### 3.3.2. Cluster II—“Cloppenburg/Oldenburg (CLOL)”

The Ger-12-16.2 poultry Cluster II “Cloppenburg/Oldenburg (CLOL)” identified in Lower Saxony displayed extraordinary outbreak dynamics, comprising a large number of poultry outbreaks (39, Supplementary Figure S2—highlighted in blue). Unlike any previously described reassortant, these disease events formed an extensive cluster in close geographical proximity, mainly affecting the districts Cloppenburg and Oldenburg (Figure 1B and Supplementary Figures S4–S6). The respective districts hold the highest poultry population density in Germany, with predominantly turkey in conjunction with domestic duck and chicken holdings. Of the 39 cases, 27 outbreaks were geographically located within a 10 km radius. In line with the temporal and spatial frame, the phylogenetic background points toward a single predicted common ancestor.

On 13 December 2016, the first outbreak of the cluster was identified in a turkey farm in Lower Saxony (AR10523/16), approximately 55 km south of the described Cluster II hotspot. Two weeks later, this virus was reported in a turkey holding located in the geographical center of the future poultry cluster. Subsequent outbreaks showing genetically analogous sequences were recorded until April 2017 (Supplementary Figure S4).

The outbreak dynamics in this geographically compact, severe eruption could not only be divided in a temporal manner but also according to genetic sequencing data. The first wave, characterized by cases from December 2016–early February 2017, affected nine turkey holdings (AR11416/16, AR11581/16, AR11632/16, AR01/17, AR43/17, AR155/17, AR395/17, AR578/17, and AR1050/17—Supplementary Figure S5, yellow coloring). Sequences from these outbreaks show identity levels of 99.83–99.97%, equaling 10–46 nucleotide differences throughout the entire genome between the individual outbreaks. The very close genetic relationship and structure of the median-joining network indicate that these outbreaks were not due to independent introductions but suggest an epidemiological linkage.

Ger-12-16.2 was again identified in samples from the same area from late February onwards, ending with the last outbreak in early April 2017. Within the second wave, 29 poultry holdings (turkey, domestic duck, and chicken) were affected, and extremely high identity levels of 99.90–100% between the singular cases were recorded, equaling 0–14 nucleotide differences throughout full-length genome sequences. The second wave holds a group of eight extremely similar viruses as common precursors (AR1807/17, AR1896/17, AR1916/17, AR1964/17, AR2350/17, AR2606/17, AR2607/17, and AR3128/17—Supplementary Figure S5, dotted circle), from which all other sequences of this wave are predicted to have evolved. Two further subclusters within the large Cloppenburg/Oldenburg Cluster II could be identified within the second wave. This includes a group of six outbreaks with larger geographical distances (AR2552/17, AR2732/17, AR2859/17, AR2921/17, AR2976/17, AR3012/17—Supplementary Figure S5, purple coloring) and a smaller group of four outbreaks within the main hotspot and in close geographical proximity (AR2374/17, AR2493/17, AR2541/17, AR2909/17—Supplementary Figure S5, orange coloring).

While the above-described data suggest tight epidemiological linkage even between farms up to 51 km distant from each other, there are also examples of the opposite: Some outbreaks of extremely close proximity showed proportionally vast genetic differences (AR2921/17 and AR2610/17, approximately 200 m airline distance and 99.81% identity levels). This is in accordance with findings that direct airborne transmissions between cases of close location distance by wind are extremely unlikely and can be largely excluded for all outbreaks in this district during the entire time of the epidemic in a detailed follow-up study (Lüder et al., manuscript in preparation/personal communication). The sequences of Cluster II showed unprecedented high similarity values between the individual poultry holdings, most likely suggesting the continuous circulation of the introduced virus within the area by direct contact (personnel) or mechanical vectors (shared machines, feed, bedding) (Lüder et al., personal communication) and evolution by point mutations (genetic drift).

### 3.4. Cluster III: Genetic Modification by Reassortment in Poultry Holdings—H5N5 Ger-12-16-N5.1 and Ger-12-16-N5.2

Alongside the portrayed H5N8 reassortants, the H5N5 subtype was identified comprising two novel H5N5 reassortants, Ger-12-16-N5.1 and Ger-12-16-N5.2 [7]. The dynamics of the H5N5 poultry outbreak have majorly differing characteristics in comparison to the previously described H5N8 reassortants [8].

#### 3.4.1. Epidemiological Data

On December 13, 2016, the H5N5 reassortant Ger-12-16-N5.1 was pinpointed in a swan (*Cygnus* sp.) in Saxony (AR10645/16). Following into February 2017, four further wild bird-derived Ger-12-16-N5.1 sequences were detected in Saxony (grey heron (*Ardea cinerea*)—AR572/17, common buzzard (*Buteo buteo*)—AR1117/17), Lower Saxony greylag goose(*Anser anser*)—AR11353/16) and Schleswig-Holstein (barnacle goose (*Branta leucopsis*)—AR11505/16).

Throughout the period 2016/17, only one outbreak in poultry, a turkey holding in Schleswig-Holstein, was caused by H5N5 viruses in Germany. Reported at the end of January 2017, the respective outbreak shed light on poultry holdings as a source of new reassortants. In addition to the reassortant virus Ger-12-16-N5.1 that was evidently circulating in wild birds in Germany, a novel reassortant Ger-12-16-N5.2, significantly varying in the NP and PB1 segments, was isolated from different barns of the same turkey holding (Cluster III SH).

**Figure 3:**
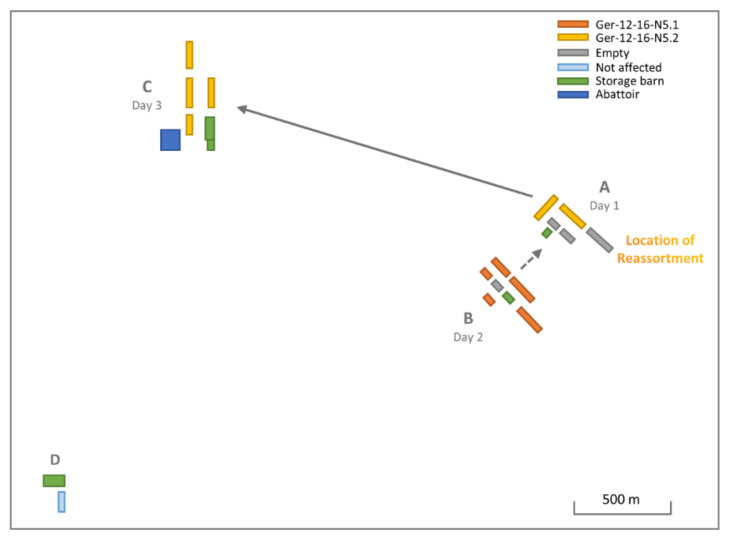
Schematic representation of the H5N5-infected poultry holding (Ger-12-16-N5.1—orange, Ger-12-16-N5.2—yellow) with view of the individual locations (A–D). Buildings are colored according to usage, dotted line indicates potential carryover and reassortment between locations, and continuous line represents definite carryover between buildings A and C. Location D was not affected.

The affected turkey rearing and fattening holding incorporated four separate locations (A–D) within a 4 km radius, each containing multiple individual barns (Figure 3). Samples from location A collected on day 1 of the outbreak (22 January 2017) tested positive for HPAIV H5N5, followed only 24 h later by a positive H5N5 test in location B (23 January 2017), located approximately 400 m from location A. Location C tested positive for H5N5 six days after the initial outbreak (28 January 2017, 2.9 km airline distance to location A). Location D was culled as a precautionary measure but showed no signs of clinical infection, and all testing remained negative. Cross-transfer of the virus between the individual locations is epidemiologically plausible, e.g., due to shared equipment, machines, and staff.

#### 3.4.2. Genetic Modification by Reassortment

In order to achieve a better understanding of the respective poultry outbreak and its connection to the wild bird cases, 17 turkey samples selected to represent all individual barns and locations of the premises were sequenced. Additionally, 12 HPAIV and LPAIV wild bird cases selected from the surrounding area and overlapping timeframe were sequenced. This allowed the genetic differentiation of both H5N5 reassortants and their assignment to the different locations (Supplementary Figures S7 and S8).

Of the 17 sequenced samples from the H5N5 poultry outbreak, only two sequences, both from location B, clustered with Ger-12-16-N5.1, while the other 15 sequences derived from samples of locations A and C were clearly identified as Ger-12-16-N5.2. Between all Ger-12-16-N5.1 sequences, identity levels of 99.67–99.98% were found. Ger-12-16-N5.2 shared similar identity levels of 99.71–100%. Multiple barns had samples of 100% identity, and even between the varying locations, A and C levels of over 99.99% were determined. Deep sequencing and thus the possibility of variant analysis allowed the identification of both H5N5 reassortant NP segments in samples from location A, pointing toward a mixed infection within the barns. However, no similar indications were found for the PB1 segments, nor could any further underlying partial or complete sequences of a corresponding LPAIV be identified within the sequenced samples from this holding. Therefore, the location of the reassortment event creating Ger-12-16-N5.2 could not be unambiguously identified.

Direct involvement of the turkey holding nevertheless remains highly suggestive because wild birds infected with this respective novel Ger-12-16-N5.2 reassortant were only pinpointed after the poultry outbreak and restricted to the surrounding area in Schleswig-Holstein until March 2017 (Supplementary Figures S8 and S9). Thereafter, the Netherlands also reported Ger-12-16-N5.2 infections in wild birds [50]. No HPAIV Ger-12-16-N5.2 reassortants were identified in other German federal states or in other countries. These observations indicate that an introduction of the reassortant from the poultry premises into the wild bird population is highly likely.

### 3.5. Cluster IV, V, and VI: Cluster Development and Distant Spread—H5N8 Ger-10-20-N8

Starting in October 2020, Germany was hit by a new wave of HPAIV cases in wild birds and outbreaks in poultry. During the course of the outbreak, numerous different subtypes, reassortants, and genotypes were identified [10]. The vast majority were assigned to the H5N8 subtype. A large number of cases in wild birds raised concerns about the putative emergence of poultry clusters.

#### Epidemiological Data and Time-Scaled Phylogeny Analysis

Indeed, from December 2020 to January 2021, a number of outbreaks of H5N8 HPAIV occurred in poultry farms, mainly turkey and two chicken farms, in the districts of Cloppenburg and Oldenburg. The analysis of the genomes from these outbreaks showed a high degree of similarities between the viruses, indicating a connected cluster (Supplementary Figure S10). Detailed analysis with time-scaled phylogeny and inferred spatial-time distribution indicates very close epidemiological links between these outbreaks (Supplementary Figure S11). The temporal scaling also allows for identifying the discharge of these viruses into the wild bird population during the course of the event (Supplementary Figure S11). Hence, the original introduction could not yet be determined.

In February and March 2021, a series of outbreaks again occurred in turkey farms in the districts of Cloppenburg and Diepholz (Supplementary Figures S12 and S13). The analysis of the genomes showed that these events were independent of the outbreaks in December and January and indicated a second incursion into the region. The genomes within the cluster showed a high degree of similarity. This again points to very close epidemiological links between these outbreaks. Both clusters show dispersal to linked wild bird cases or poultry outbreaks in more distant districts. The continuous presence of HPAIV viruses in close temporal and spatial proximity increases the risk of spill-over to the wild bird population.

The potentially devastating role of moving infected poultry became clearly evident in March 2021, when an HPAI outbreak of subtype H5N8 was confirmed on a farm raising laying hens for live, ambulant sale. The index farm sold layer chickens to customers, partly by traveling sellers, which was responsible for over 100 secondary poultry outbreaks in five federal states [51]. The time-scaled phylogeny and inferred spread of the viruses suggest a common ancestor for Cluster V and VI (Supplementary Figure S14). The connection between clustered areas and their directed spread is summarized in Supplementary Figure S15. This uncontrolled dissemination of HPAIV viruses by traveling sellers is not only a threat to the poultry industry with high commercial losses. In this case, it was mainly small and medium-sized poultry farms that were affected. This case takes on a special significance since the analysis of the total genomes from the index case, its second location, and from flocks infected by the live sale revealed a mutated PB2 gene, harboring an E627K amino acid exchanged known to increase host specificity toward mammals and therefore elevate the risk of zoonotic infections.

**Figure 4:**
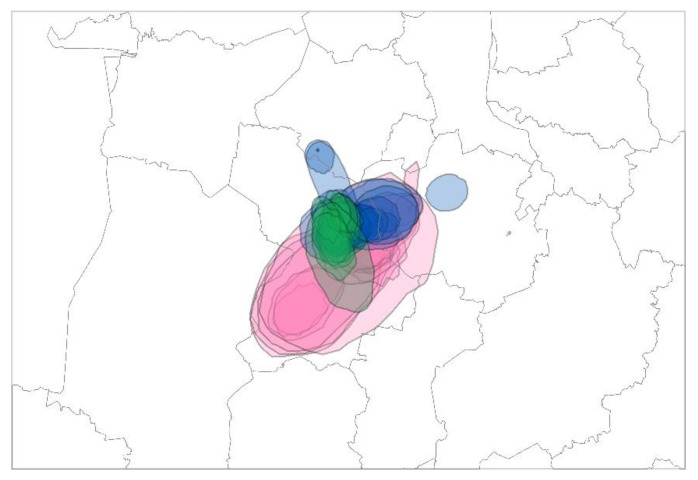
Areas of three poultry clusters identified in Lower Saxony during two seasonal outbreaks, 2016/2017 and 2020/2021. Cluster II CLOL Ger-12-16-N8.2 (2016/2017), blue; Cluster V CL Ger-10-20-N8 (2021), green; Cluster IV CL Ger-10-20-N8 (2020/2021), pink.

The inferred areas of identified clusters in two seasonal outbreaks, 2016/2017 and 2020/2021, show a high degree of overlap with a focus on three municipalities in Lower Saxony known to harbor the highest density of poultry holdings in Germany (Figure 4).

## 4. Discussion

The tendency for frequent reassortment and the subsequently reported extensive capability in attaining novel genome segments are a defining characteristic of HPAIV of the gs/GD lineage clade 2.3.4.4b in comparison to other gs/GD clades [18]. Represented by numerous reassorted genotypes across Asia, North America, Europe, and Africa since 2014, clade 2.3.4.4b has spread unceasingly and unprecedentedly, with reassortment expected to translate into phenotypic features that are accessible to selective forces whereby shaping and optimizing viral fitness. In line with these findings, the European HPAI H5Nx clade 2.3.4.4b epidemics in 2016/2017 and 2020/2021 have revealed multiple subtypes and reassortants over the course of the events [10,26]. This is also mirrored in the German situation with the identification of three reassorted HPAI H5N8 viruses, two reassorted HPAI H5N5 viruses in 2016–2017, and an additional new reassorted H5N6 virus in 2018 [8]. A further novel HPAI clade 2.3.4.4b H5N8 reassortant was detected in Europe in January 2020 [48]. These findings shine a light on the importance of accurate full-length genome sequencing to dissect outbreaks for the precise identification of reassortants.

The detailed phylogenetic and spatiotemporal analyses of the HPAIV clade 2.3.4.4b epidemic allow insights into wild bird and poultry interactions, clustering, continuous circulation of HPAIV and genetic drift, as well as poultry holdings as a ground for reassortment.

Passive and active surveillance of the wild bird population has been shown to be of utmost importance. Here, due to the proportionally small number of wild bird cases sequenced, some gaps still exist within the analyses. Surveillance of poultry holdings, especially commercial farms, is stipulated by the law, leading to more intensive sampling and strict mortality rate observation. Thus, the level of disease detection in poultry holdings is far higher than in the wild bird population, where positive sampled birds are the “tip of the iceberg” [52]. In addition, as in the most recent HPAIV clade 2.3.4.4b epidemic in the winter of 2019/2020 [37,48], some wild bird species, such as dabbling ducks, and in particular the very common mallard, do not necessarily show higher mortality rates, thus interfering with passive surveillance. Although only a proportionally few wild birds were sequenced in this study, their geographic and temporal distribution with emphasis on areas of great interest (both clusters and the surrounding area of the infected H5N5 outbreak) covers Germany to provide a respective insight into the distribution and prevalence of the individual reassortants.

Geographical vicinity does not automatically result in genetic unity. Similar to the 2017–2018 H5N6 outbreaks described in the Netherlands [53], genetic distinctions can help to establish the differentiation between farm-to-farm transmissions and separate introductions from wild birds. Many examples can be given for close geographical proximity, yet infection with diverging reassortants: In Lower Saxony cluster shows multiple Ger-11-16 poultry outbreaks and wild bird cases within the same time period as the first wave of the Ger-12-16.2 cluster and within the same districts, in some instances with as little as 5 km distance between cases of both reassortants. Distinctions within an individual reassortant can likewise be pinpointed; for instance, the Ger-12-16.2 poultry cluster described in the federal state of Brandenburg, with only a 35 km distance between two genetically distinct cases (AR1464/17 in comparison to AR877/17, AR681ff/17).

All reassortants were identified in both wild bird cases and poultry outbreaks, except, for example, no wild bird sequences were detected within the large Ger-12-16.2 reassortant Cloppenburg/Oldenburg cluster. As previously described, this was the largest cluster of the epidemic, accounting for 39 outbreaks in poultry holdings. Despite sequencing a comprehensive subset of wild birds, both in close geographical and temporal proximity and throughout other federal states, no genetically associated wild bird-derived virus was identified. Due to the phylogenetic analyses conducted, it is clear that, as a minimum, two individual virus entries into the area must have taken place, at the very least for the first and second wave of the outbreak. Although infected wild birds evidently play a wide-ranging role in the dissemination of AIV, farm-to-farm transmissions can likewise trigger major outbreaks and promote and sustain the continuous local circulation of the virus by direct or indirect contact between poultry holdings. In the Cloppenburg/Oldenburg cluster, the extremely high similarity rates amid individual holdings (up to 100% identity) permit the assumption of direct connections between poultry premises. Identifying critical contact points by field epidemiological investigations and compliance with high hygiene standards are of utmost importance to curb farm-to-farm transmissions, thus preventing continuous local spread. Moreover, if farm-to-farm transmission is possible, re-introduction of HPAIV from affected poultry farms into the wild bird population must also be expected and should be prevented to avoid further reassorting and spreading.

Although reassortment events at common wild bird molting and breeding grounds have been observed [11,14,18,22], local reassortment within the resident wild bird population or potentially even within a poultry holding also is conceivable. Due to the temporal and genetic manner of the H5N5 outbreak described here, the respective premises infected with both H5N5 reassortants was perchance the location of the Ger-12-16-N5.2 reassortment event. It is conceivable that the turkey holding was previously infected with a local European LPAI virus without displaying clinical symptoms, and after the introduction of the Ger-12-16-N5.1 virus to location B and the highly plausible transmission of Ger-12-16-N5.1 to location A via direct contact (collective workers, machines, feed or bedding), the reassortment event occurred within the barns at location A of the affected holding. Although location A showed increased mortality 24 h before location B, the new reassortment may have affected the virulence of the virus, or the poultry at location A were potentially weakened by the presumed LPAIV infection. Further transmission to location C, showing clinical signs and high mortality 5 days post identification of infection at location A, was established, substantiating the possibility of transmission between the individual sites of the premises by direct contact. In addition, as the Ger-12-16-N5.2 reassortant had never been identified anywhere prior to the German poultry case, a reassortment event in the respective holding appears even more likely. After the poultry outbreak, the Ger-12-16-N5.2 reassortant was identified in wild birds in close vicinity to the holding and throughout Schleswig-Holstein. These findings suggest an introduction from the infected poultry premises into the environment, thus primarily infecting the local wild bird population and contributing toward the further distribution of the novel reassortant, later identified in the Netherlands [51].

## 5. Conclusions

The HPAI clade 2.3.4.4b 2016–2017 and 2020–2021 epidemics represent some of the most severe poultry and wild bird disease events in Germany, comprising several subtypes and reassortants. The conducted phylogeographic analyses permitting the detailed genetic tracing of cases highlighted the varying outbreak dynamics and role of poultry in the course of spread and genesis of reassortants. Hotspots harboring large poultry clusters were described, in which the role of poultry in farm-to-farm transmission via direct contact and continuous local circulation between poultry holdings in dense farming areas with no directly established wild bird connection was identified. It also became evident that poultry can serve as a source of reassortment, demonstrated by an event of H5N5 reassortment within a single holding after a potential mixed infection with LPAI and HPAI viruses, followed by subsequent introduction of the novel reassorted virus into the local wild bird population. Full-length genome sequencing is of utmost importance to dissect AIV outbreaks thoroughly and understand the dynamics and distribution of individual reassortants. Although passive wild bird surveillance stays indispensable, active wild bird sampling would benefit the evaluation and allow accurate statements on the origin and distribution of predecessor AIV.

## Figures and Tables

**Table 1:** Metadata of reassortants regarding subtype, first and last identification date, number of overall sequenced cases/outbreaks followed by poultry and wild bird sequence counts.

Reassortant	Subtype	First Date	Last Date	Total	Poultry	Wild
**Ger-11-16-N8**	H5N8	7 November 2016	22 August 2017	34	25	9
**Ger-12-16-N8.1**	H5N8	1 January 2017	24 February 2017	8	2	6
**Ger-12-16-N8.2**	H5N8	13 December 2016	9 May 2017	71	61	10
**Ger-12-16-N5.1**	H5N5	13 December 2016	23 January 2017	7	2	5
**Ger-12-16-N5.2**	H5N5	22 January 2017	9 March 2017	22	15	7
**Ger-10-20-N8**	H5N8	26 October 2020	20 July 2021	161	122	39
**Ger-02-21-N8**	H5N8	26 February 2021	10 March 2021	3	3	0
**Ger-03-21-N8**	H5N8	2 March 2021	2 March 2021	1	1	0
**Ger-10-20-N5**	H5N5	26 October 2020	9 November 2020	2	1	1
**Ger-12-20-N3**	H5N3	14 December 2020	26 January 2021	5	0	5
**Ger-02-21-N4**	H5N4	17 February 2021	17 February 2021	2	0	2
**Ger-02-21-N1**	H5N1	17 February 2021	17 July 2021	9	4	5

## Data Availability

All sequences were made publicly available in the GISAID database (www.gisaid.org). Associated data and underlying source data are available in the https://zenodo.org/ repository under doi:10.5281/zenodo.6826696.

## Supplementary Materials

The following supporting information can be downloaded at: https://www.mdpi.com/article/10.3390/v14091849/s1, Figure S1: Ger-11-16 median-joining network, Figure S2: Ger-12-16 median-joining network, Figure S3: Cluster I spatial-time phylogeography, Figure S4: Cluster II MCC phylogeny, Figure S5: Cluster II graphic representation and median-joining network, Figure S6: Cluster II spatial-time phylogeography, Figure S7: Cluster III median-joining network, Figure S8: Cluster III MCC phylogeny, Figure S9: Cluster III spatial-time phylogeography, Figure S10: Cluster IV MCC phylogeny, Figure S11: Cluster IV spatial-time phylogeography, Figure S12: Cluster V MCC phylogeny, Figure S13: Cluster V spatial-time phylogeography, Figure S14: Cluster V + VI MCC phylogeny, Figure S15: Cluster V + VI spatial-time phylogeography.
