# Supplementary material for: Connect to Protect: Dynamics and Genetic Connections of Highly Pathogenic Avian Influenza Outbreaks in Poultry from 2016 to 2021 in Germany
Source: Viruses. 2022 Aug 23;14(9):1849. doi: 10.3390/v14091849 (PMC9502251; doi:10.3390/v14091849)
Supplement: Supplementary file 1 [file viruses-14-01849-s001.zip › viruses-1865923-supplementary.pdf]

Supplemental Material: "Dynamics and Genetic Connections of Highly Pathogenic Avian Influenza Outbreaks in Poultry from 2016 to 2021 in Germany"

Figure S1. Median-joining network comprising all Ger-11-16 genomes. Stars highlight outbreaks/cases described in the main text. Schematic representation of Ger-12-16 virus shown according to phylogenetic results as previously described (Pohlmann et al. 2018).

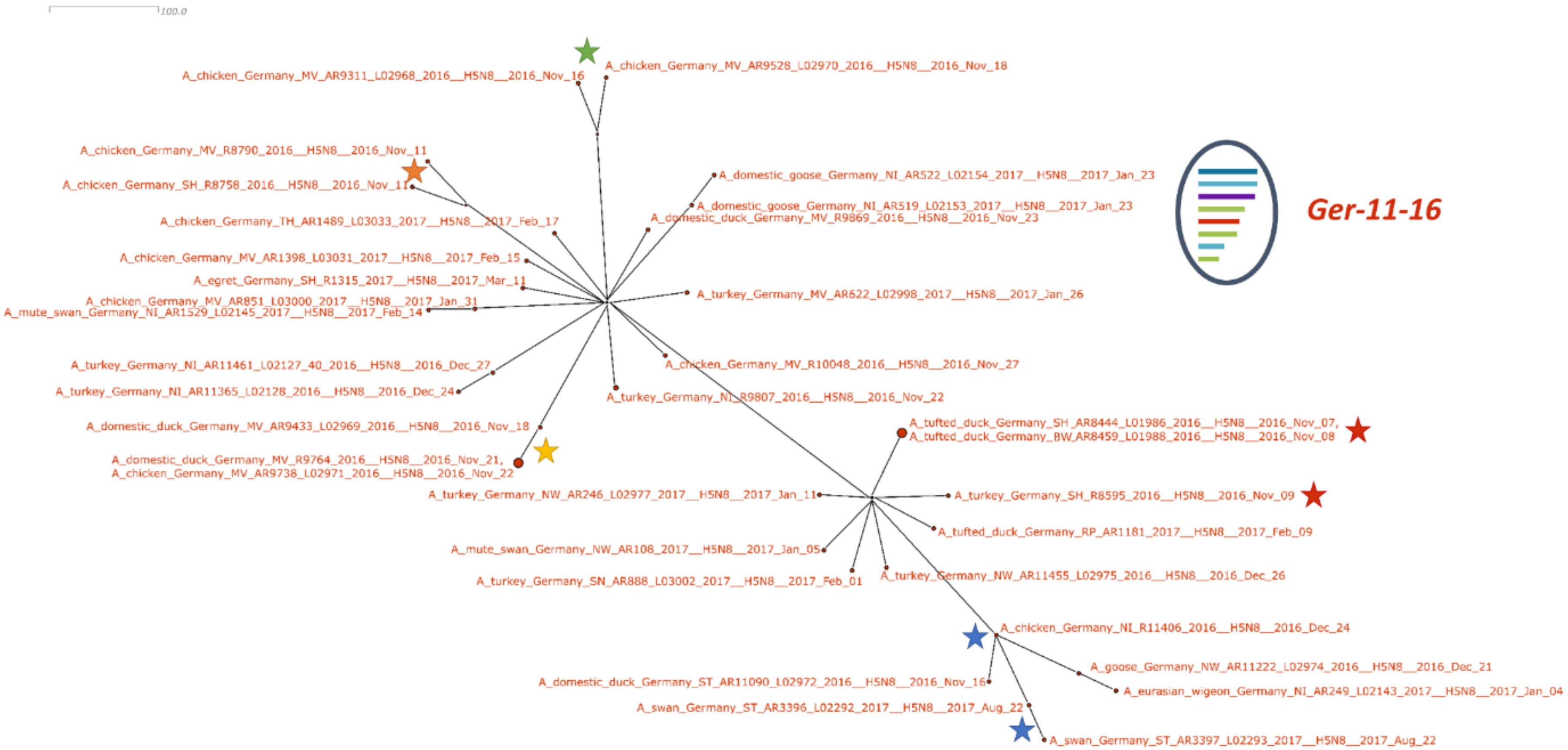

Figure S2. Median-joining network comprising all Ger-12-16 genomes (Ger-12-16.1 – light blue, Ger-12-16.2 – dark blue). Individual cases of Cluster I BB Ger-12-16-N8.2 (2017) highlighted in red, purple, yellow and green as described in the main text. Cluster II CLOL Ger-12-16-N8.2 is highlighted in blue. Schematic representation of Ger-12-16 virus shown according to phylogenetic results as previously described (Pohlmann et al. 2018).

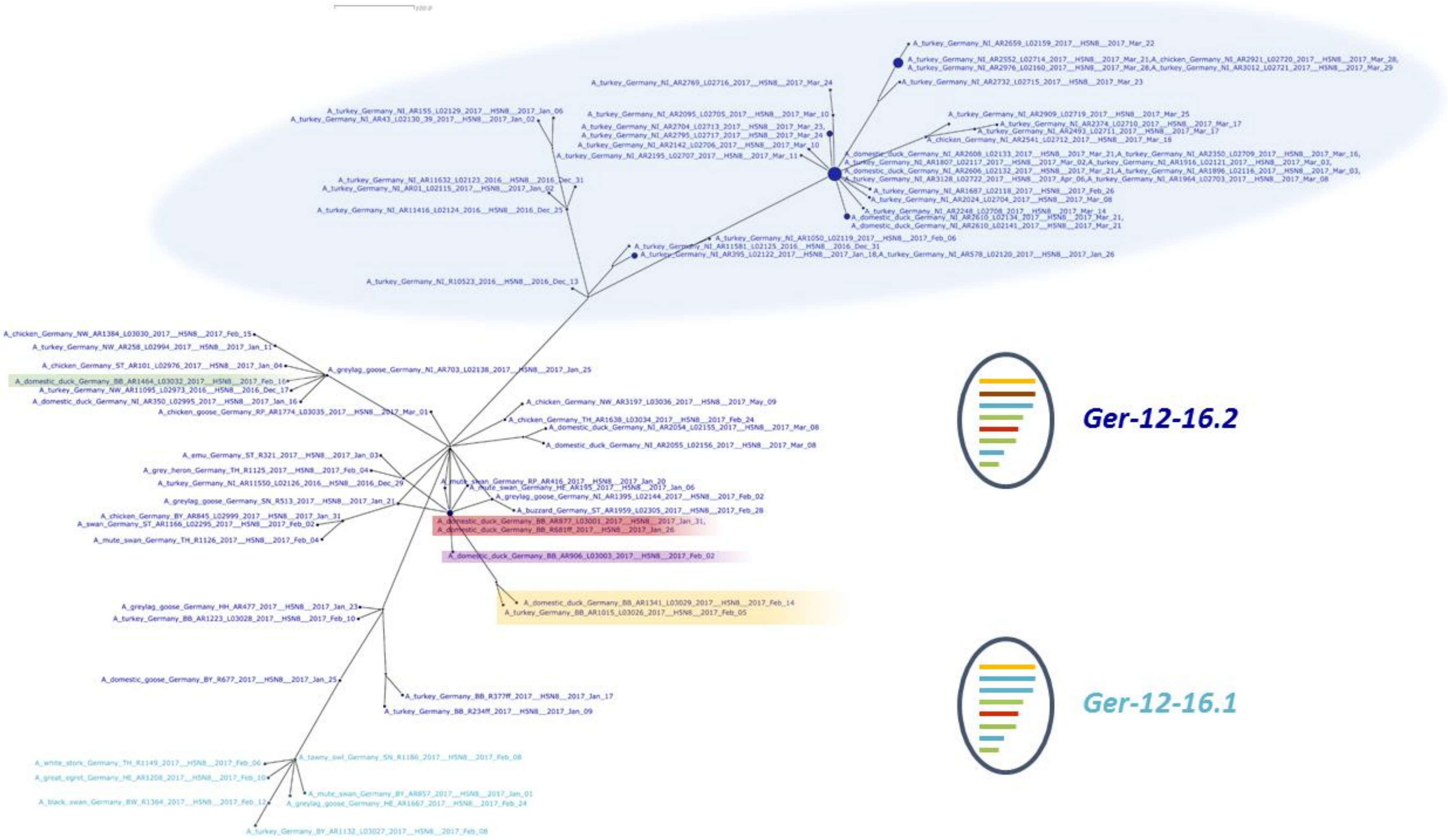

Figure S3. Connected cluster areas (polygons) with their directed spread (arrows) of HPAIV genomes Cluster I BB Ger-12-16-N8.2 (2017) inferred by spatial-time phylogeography. Cluster shown magnified as detail on the right.

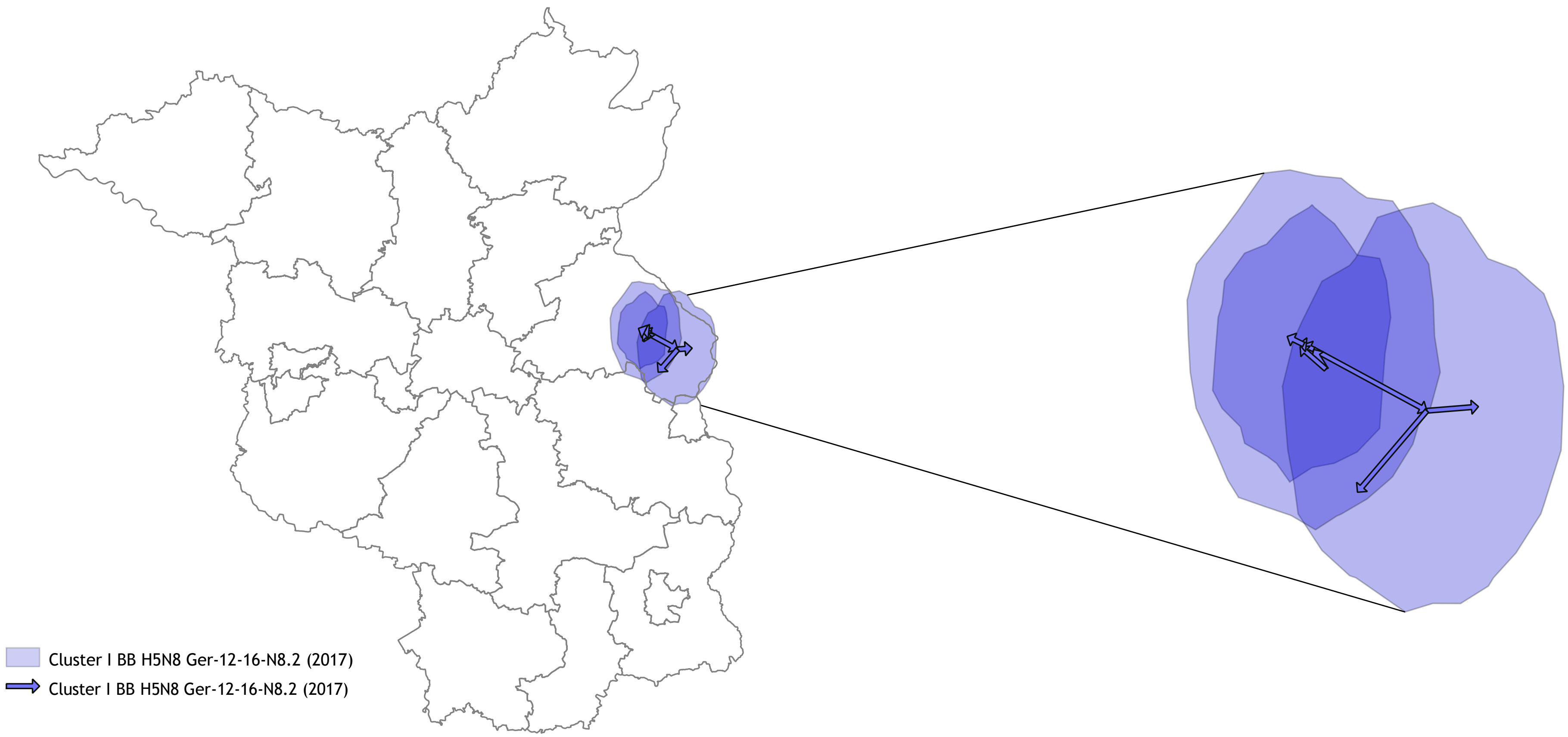

Figure S4. Time-scaled maximum clade credibility (MCC) phylogeny of concatenated Ger-12-16-N8 genomes in Cluster II CLOL. Node bars indicate 95% highest posterior density (HPD). Posterior probabilities are given at branch level.

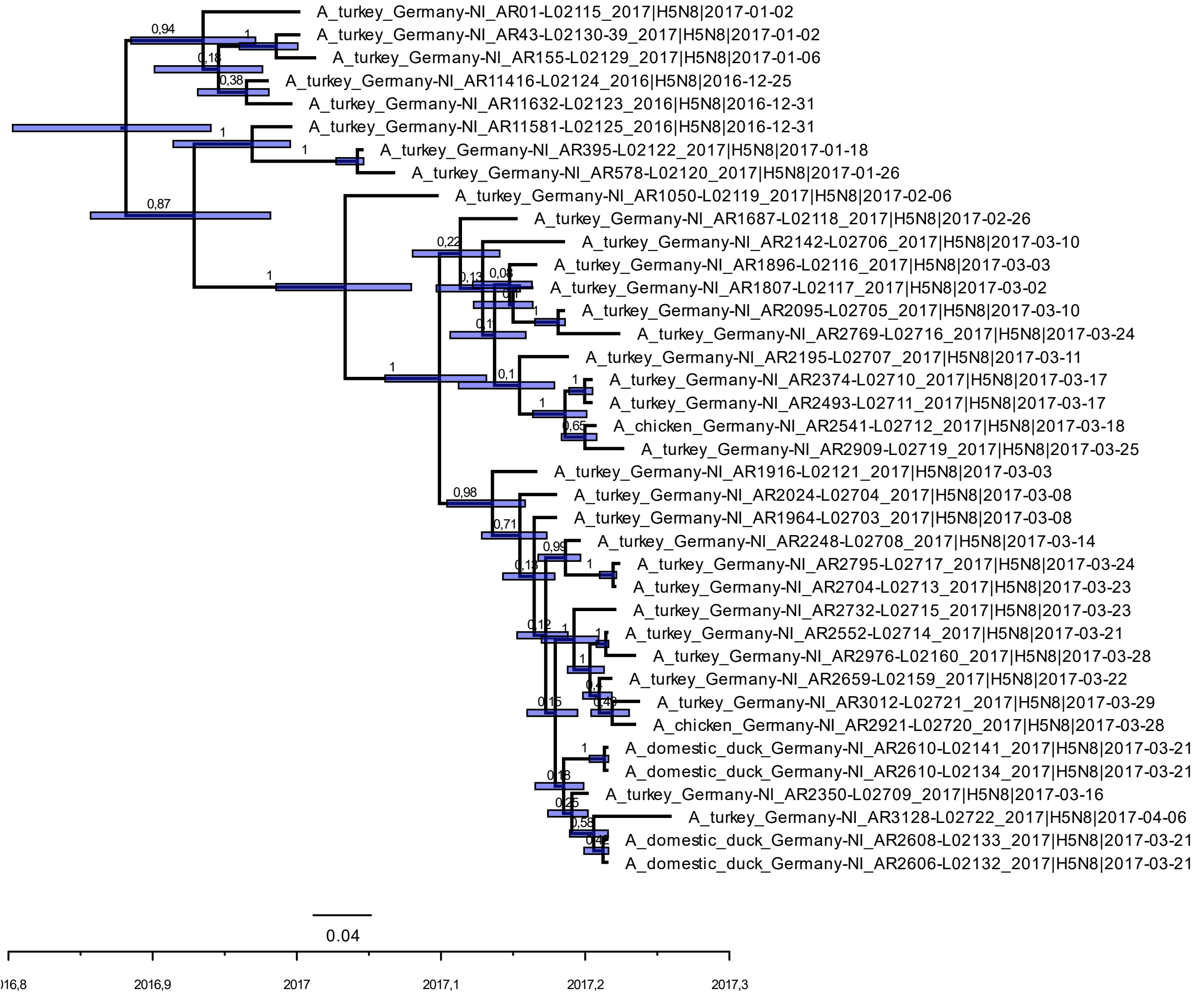

Figure S5. Graphic representation of Cluster II CLOL Ger-12-16-N8.2 (2016/2017). Colors are concurrent between Panels A and B. Panel A: Median-joining network extract of respective cluster, distinguishable between first and second wave and color-coded according to related subsets. Panel B: Schematic view of affected poultry holdings. For reasons of anonymity, no explicit locations are shown and distances are not proportional. Dotted circles – central cases, dotted lines – predicted connections according to median-joining network, continuous lines – definite direct connections.

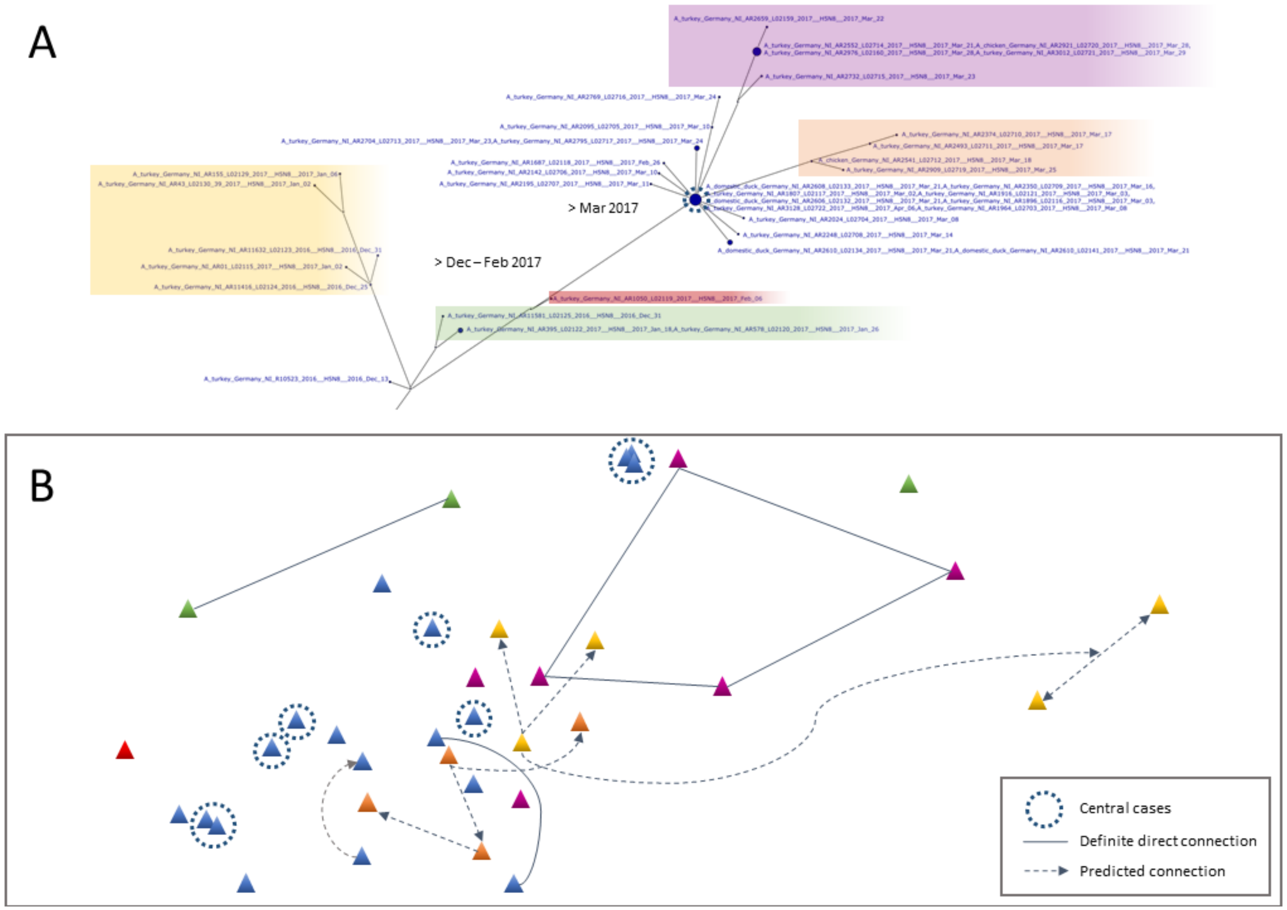

Figure S6. Connected cluster areas (polygons) with their directed spread (arrows) of HPAIV genomes Cluster II CLOL Ger-12-16-N8.2 (2016/2017) inferred by spatial-time phylogeography. Cluster shown magnified as detail.

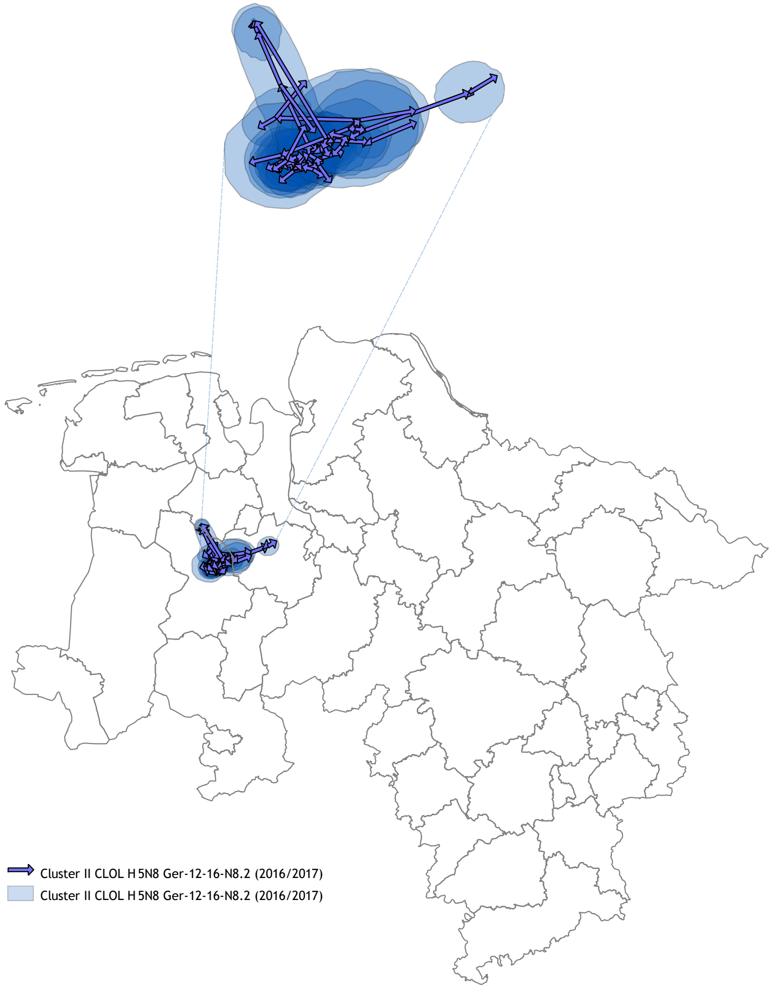

Figure S7: Median-joining network comprising all Ger-12-16-N5 genomes (Ger-12-16-N5.1 – orange, Ger-12-16-N5.2 – yellow). Stars are allocated to the respective sequences according to the location within the poultry holding. Schematic reassortant Ger-12-16-N5 viruses shown according to phylogenetic results as previously described (Pohlmann et al. 2018).

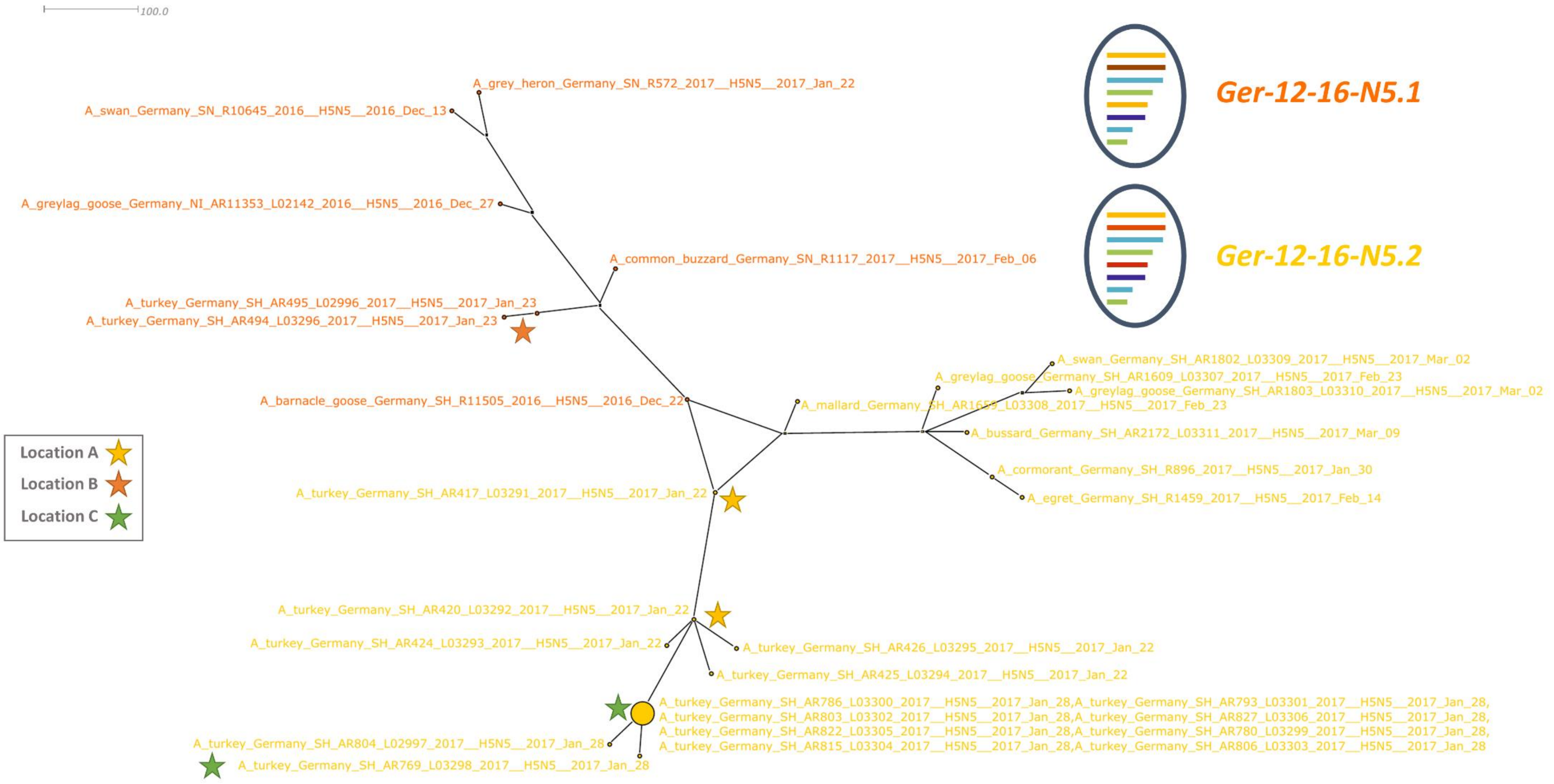

Figure S8: Time-scaled maximum clade credibility (MCC) phylogeny of concatenated Ger-12-16-N5 genomes (Ger-12-16-N5.1, Ger-12-16-N5.2) genomes. Node bars indicate 95% highest posterior density (HPD). Posterior probabilities are given at branch level.

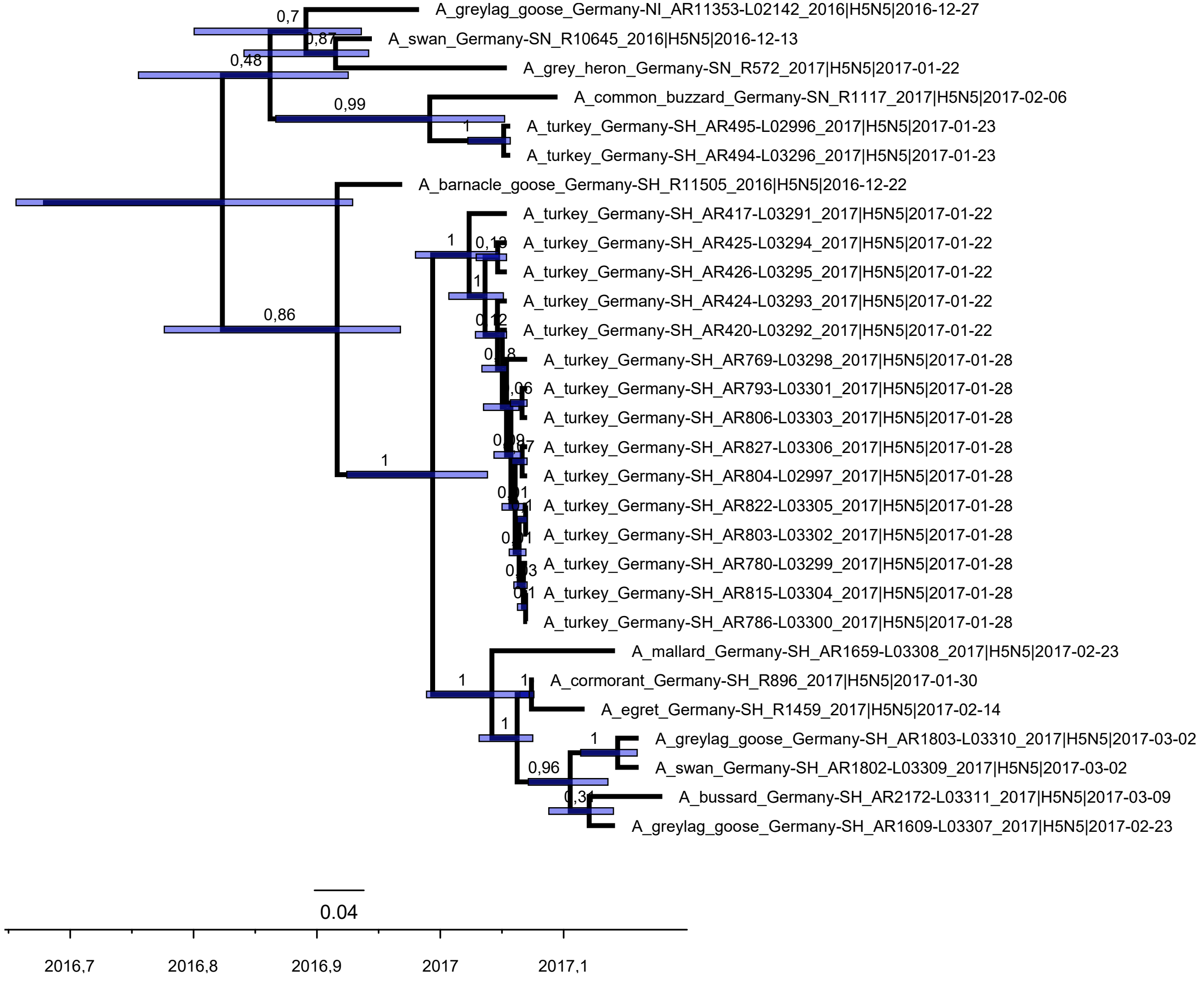

Figure S9. Connected cluster areas (polygons) with their directed spread (arrows) of HPAIV genomes Cluster III SH Ger-12-16-N5 (Ger-12-16-N5.1 – orange, Ger-12-16-N5.2 – yellow) inferred by spatial-time phylogeography. Wild bird cases are marked with stars, poultry outbreaks with circles.

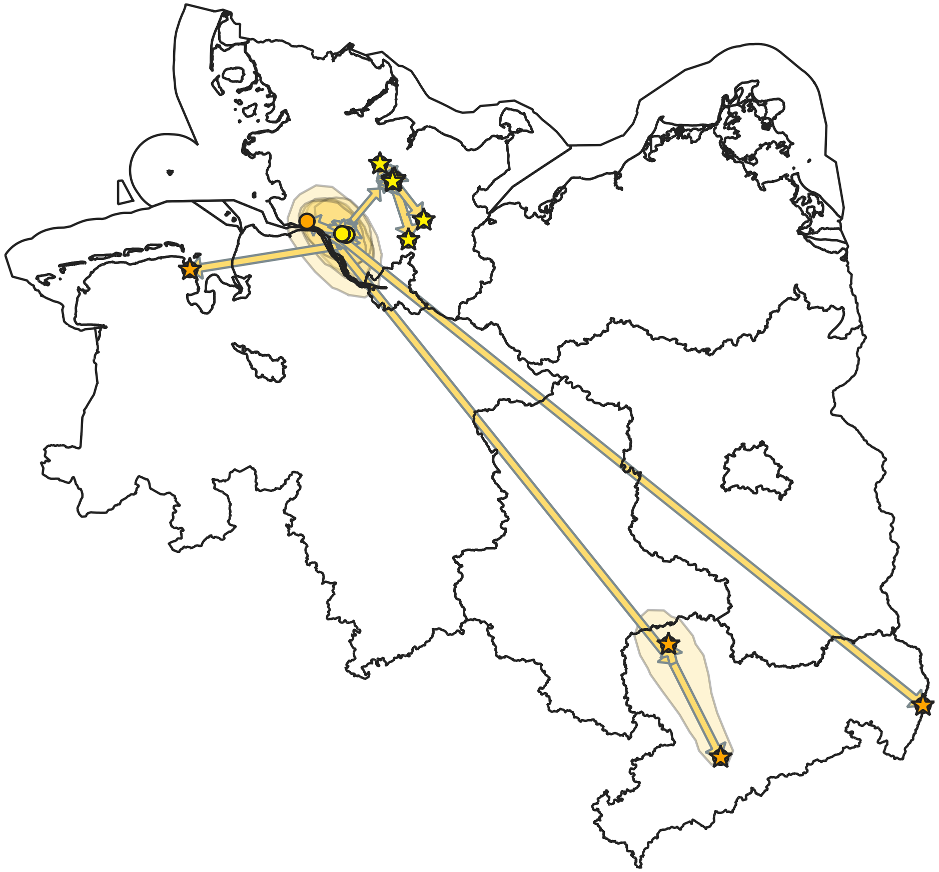

Figure S10: Time-scaled maximum clade credibility (MCC) phylogeny of concatenated Cluster IV CL Ger-10-20-N8 (2020/2021) genomes. Node bars indicate 95% highest posterior density (HPD). Posterior probabilities are given at branch level.

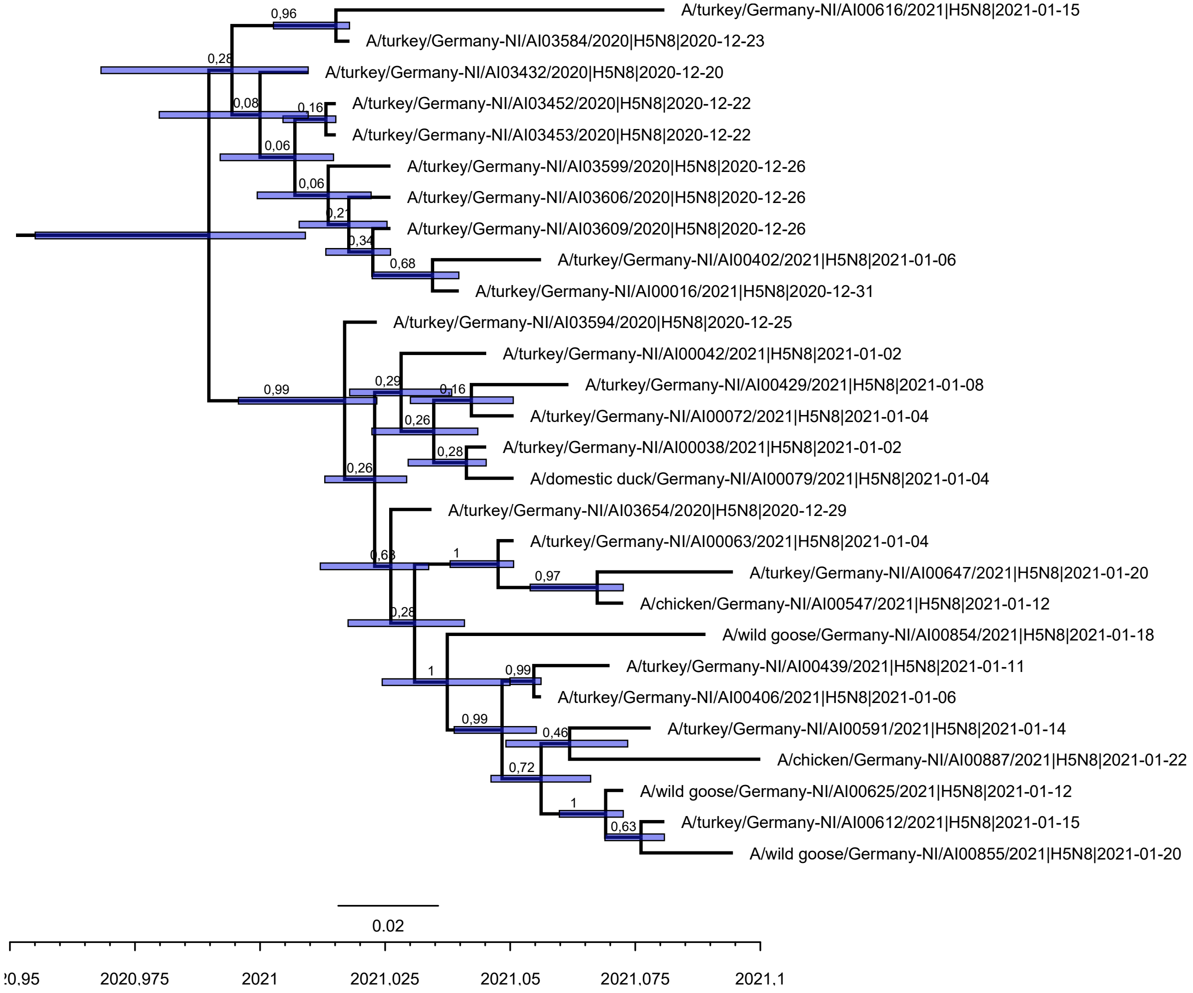

Figure S11. Connected cluster areas (polygons) with their directed spread (arrows) of HPAIV genomes Cluster IV CL Ger-10-20-N8 (2020/2021) inferred by spatial-time phylogeography.

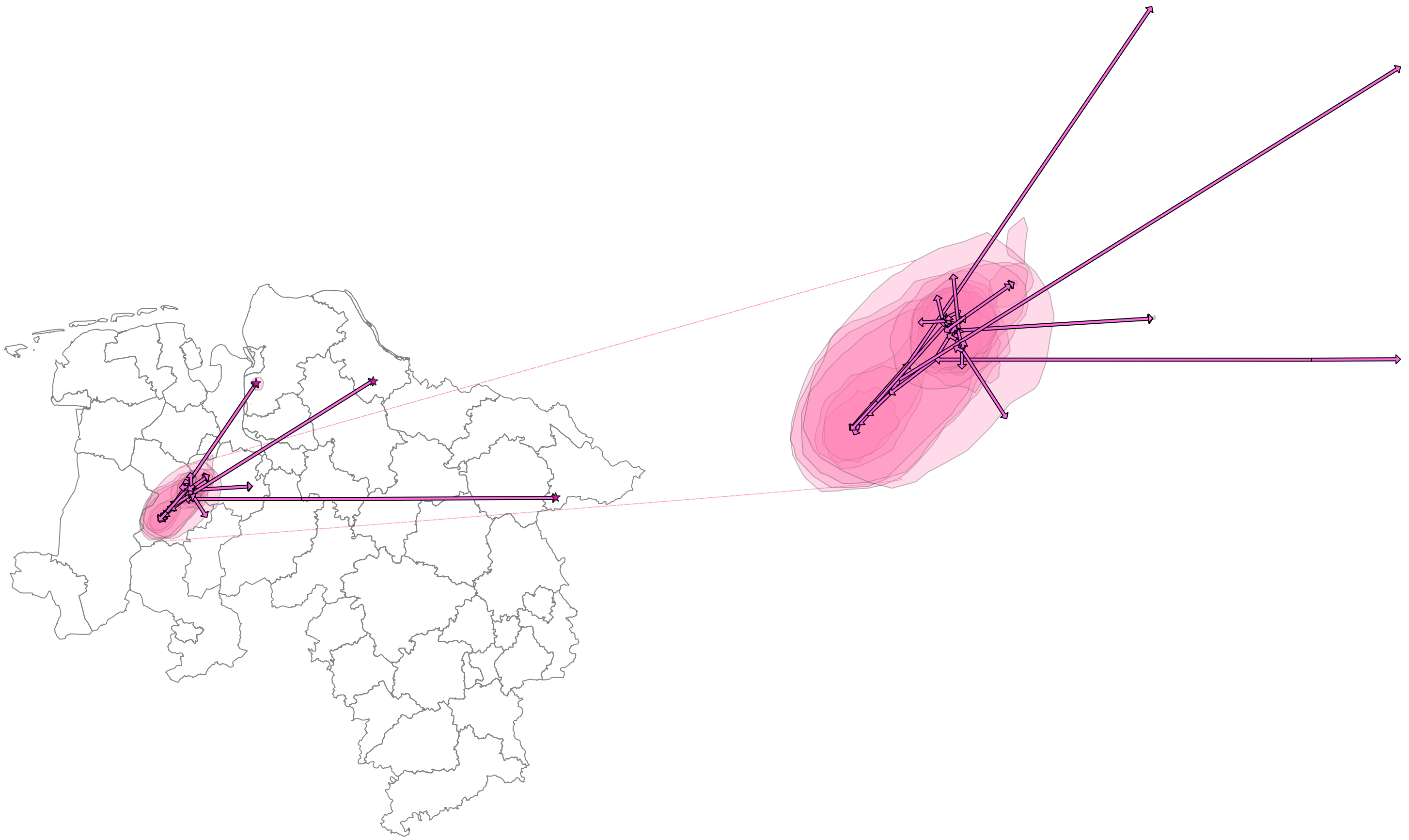

Figure S12: Time-scaled maximum clade credibility (MCC) phylogeny of concatenated Cluster V CL Ger-10-20-N8 (2021) genomes. Node bars indicate 95% highest posterior density (HPD). Posterior probabilities are given at branch level.

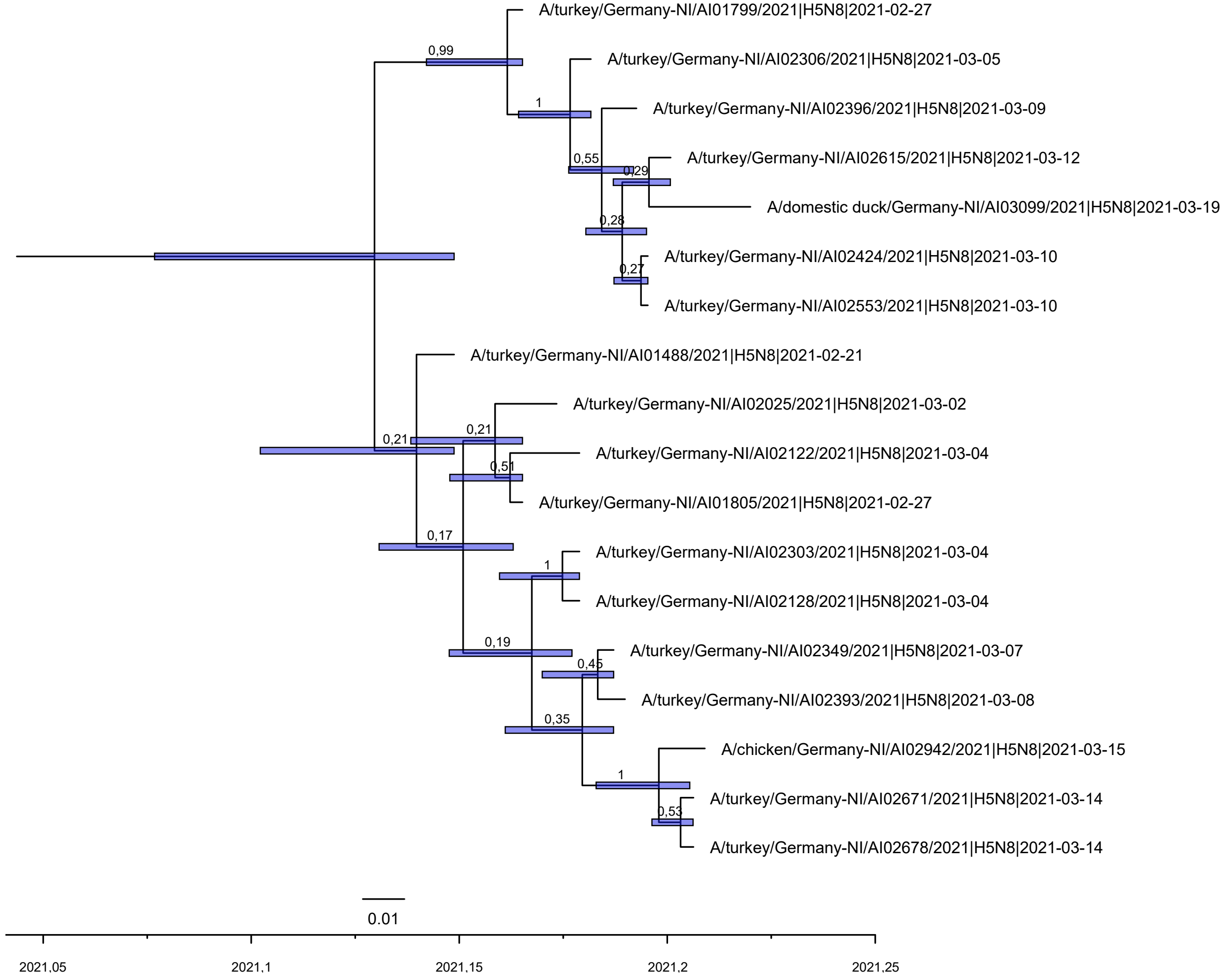

Figure S13. Connected cluster areas (polygons) with their directed spread (arrows) of HPAIV genomes Cluster V CL Ger-10-20-N8 (2021) inferred by spatial-time phylogeography.

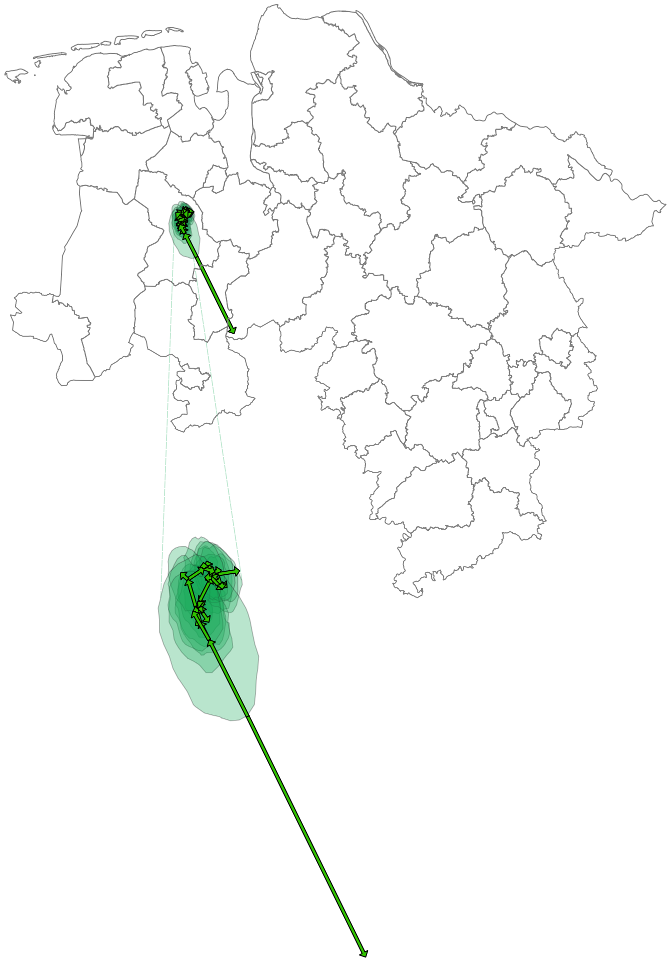

Figure S14: Time-scaled maximum clade credibility (MCC) phylogeny of concatenated Ger-10-20-N8 Cluster V + VI CL Ger-10-20-N8 (2021) genomes. Node bars indicate 95% highest posterior density (HPD). Posterior probabilities are given at branch level.

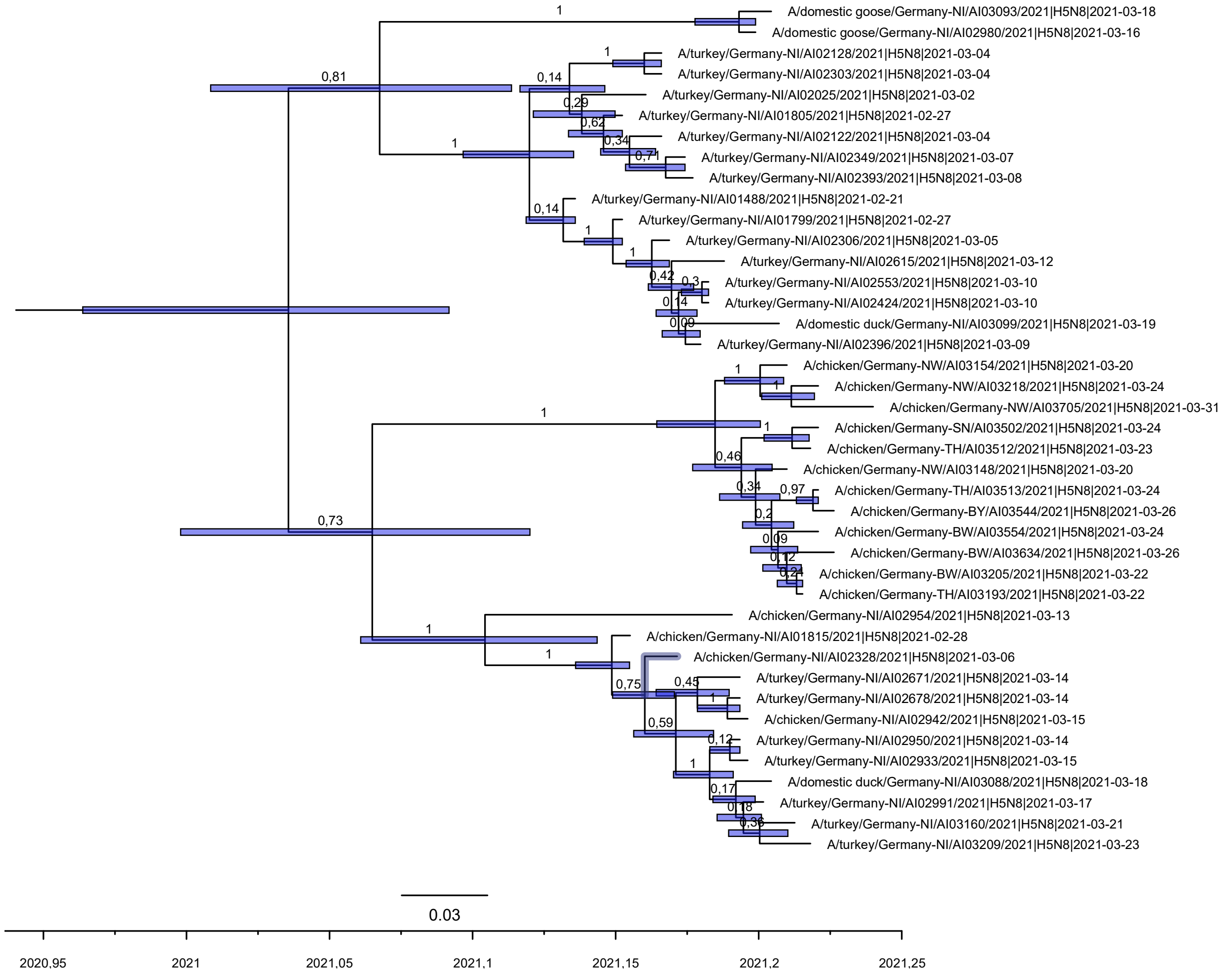

Figure S15. Connected cluster areas (polygons) with their directed spread (arrows) of HPAIV genomes Cluster V + VI CL Ger-10-20-N8 (2021) inferred by spatial-time phylogeography.

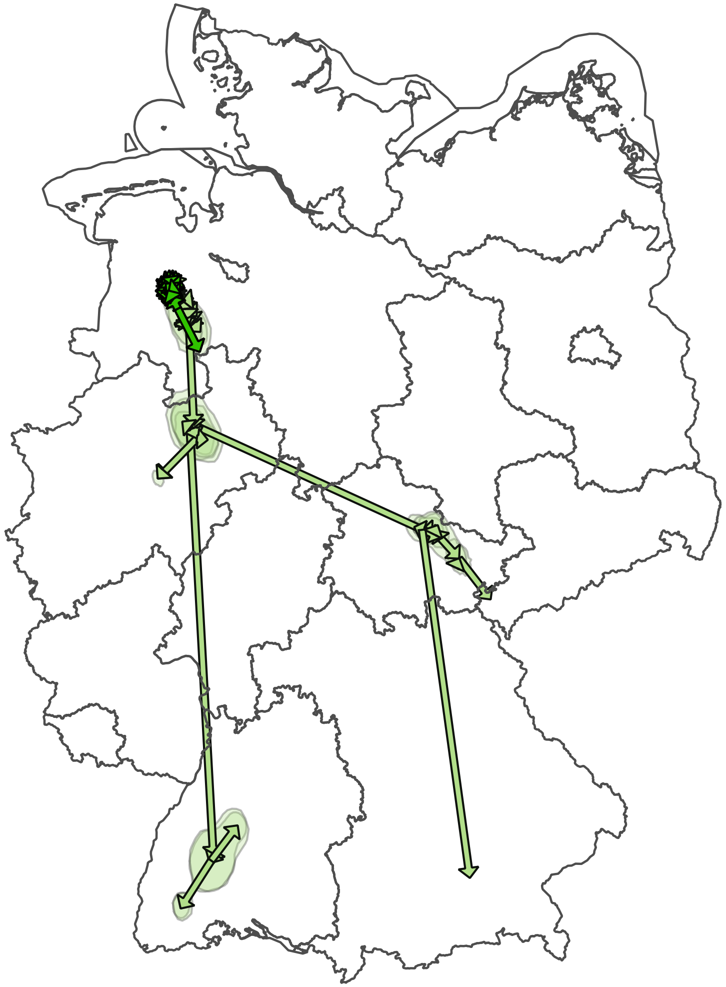

References

Pohlmann, A., E. Starick, C. Grund, D. Höper, G. Strebelow, A. Globig, C. Staubach, F. J. Conraths, T. C. Mettenleiter, T. Harder and M. Beer (2018). "Swarm incursions of reassortants of highly pathogenic avian influenza virus strains H5N8 and H5N5, clade 2.3.4.4b, Germany, winter 2016/17." Sci Rep 8(1): 15.
